# Protein–Solvent Interface Controls Proton-Coupled Reactivity in Cryptochrome 4a

**DOI:** 10.1021/jacs.6c09345

**Published:** 2026-07-10

**Authors:** Jiate Luo, Matthew Tremblay, Jonathan Hungerland, Ilia A. Solov’yov, Joseph E. Subotnik, Sharon Hammes-Schiffer

**Affiliations:** † Department of Chemistry, 6740 Princeton University, Princeton, New Jersey 08544, United States; ‡ Institut für Physik, 11233 Carl von Ossietzky-Universität Oldenburg, Carl-von-Ossietzky Str. 9−11, Oldenburg D-26129, Germany; ¶ Research Center for Neurosensory Science, Carl von Ossietzky Universität Oldenburg, 26111 Oldenburg, Germany; § Center for Nanoscale Dynamics (CENAD), Institute of Physics, Carl von Ossietzky Universität Oldenburg, 26129 Oldenburg, Germany

## Abstract

Cryptochrome 4a (Cry4a) is a leading candidate for the radical pair-based magnetoreceptor proposed to enable avian navigation. Following photoexcitation, electron transfer along a tryptophan tetrad generates radical pairs whose recombination dynamics are thought to underlie magnetic sensitivity. However, competing proton-related reactions that may modulate these spin-selective processes remain poorly understood. Here, we combine classical molecular dynamics and quantum mechanical/molecular mechanical free energy simulations to investigate deprotonation of terminal tryptophan radical cations and a potential proton-coupled electron transfer (PCET) pathway involving a surface-exposed tyrosine. We find that limited solvent accessibility of the third tryptophan significantly suppresses its effective deprotonation, whereas the fourth tryptophan is more readily deprotonated despite similar intrinsic proton transfer thermodynamics. In addition, we identify a multisite PCET pathway in which electron transfer from tyrosine to the fourth tryptophan radical cation is coupled to proton transfer from tyrosine to interfacial water, with a free energy barrier consistent with sub-microsecond kinetics. These results demonstrate that proton transfer and PCET reactions at the protein–solvent interface can compete kinetically with radical pair recombination, thereby providing alternative pathways that may influence magnetic sensitivity. This work establishes a mechanistic framework for probing proton-coupled processes in cryptochrome-based magnetoreception.

## Graphical abstract

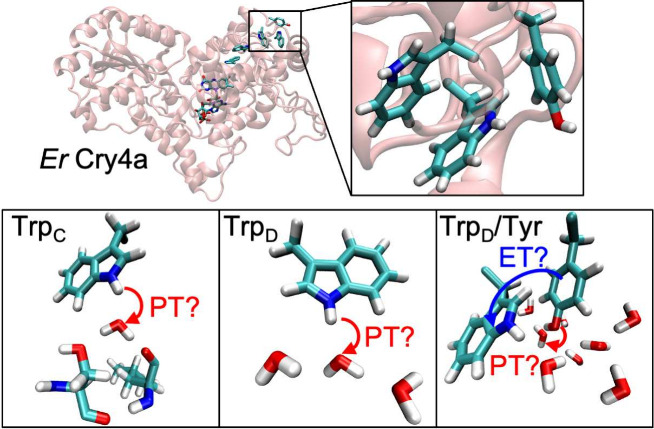

Migratory birds can navigate directionally over thousands of kilometers, yet the origin of this remarkable “compass sense” remains obscure.^1–3^ A working hypothesis is based on light-dependent magnetoreception mediated by cryptochrome, a flavoprotein found in avian retinas.^4–18^ Within this hypothesis, cryptochrome 4a (Cry4a), which tightly binds the blue-light chromophore flavin adenine dinucleotide (FAD), has emerged as the leading candidate for the magnetoreceptor.^19–25^ Moreover, recent *in vitro* photochemistry experiments have shown that Cry4a from the night-migratory European robin (*Erithacus rubecula*, *Er*) exhibits stronger magnetic sensitivity than those from nonmigratory species.^19^ Thus, *Er*Cry4a provides an important platform for probing the molecular underpinnings of avian magnetoreception.

The radical pair mechanism provides a biophysical basis for magnetic field effects in the photochemical reactions occurring in Cry4a.^5,19,26–32^ Upon blue-light excitation of FAD, sequential electron transfer along a tryptophan tetrad composed of four Trp_X_ residues (X = A, B, C or D, corresponding to W395, W372, W318 or W369, respectively) to the flavin generates radical pairs [FAD^•–^ Trp_X_H^•+^], denoted by RP_X_ (Figure 1).^19,33–37^ Within the paradigm of a radical pair mechanism, an external magnetic field modulates the efficiency of singlet–triplet interconversion as driven by hyperfine interactions and, in turn, alters the kinetics of spin-selective reactions and the product yields of various reaction pathways.^5,27,28,30,38–42^ Experimental and theoretical studies suggest that magnetoreception in *Er*Cry4a may be achieved by a “composite” radical pair arising from rapid interconversion between RP_C_ and RP_D_ with comparable equilibrium populations.^19,26,33,35,43,44^

**Figure 1:**
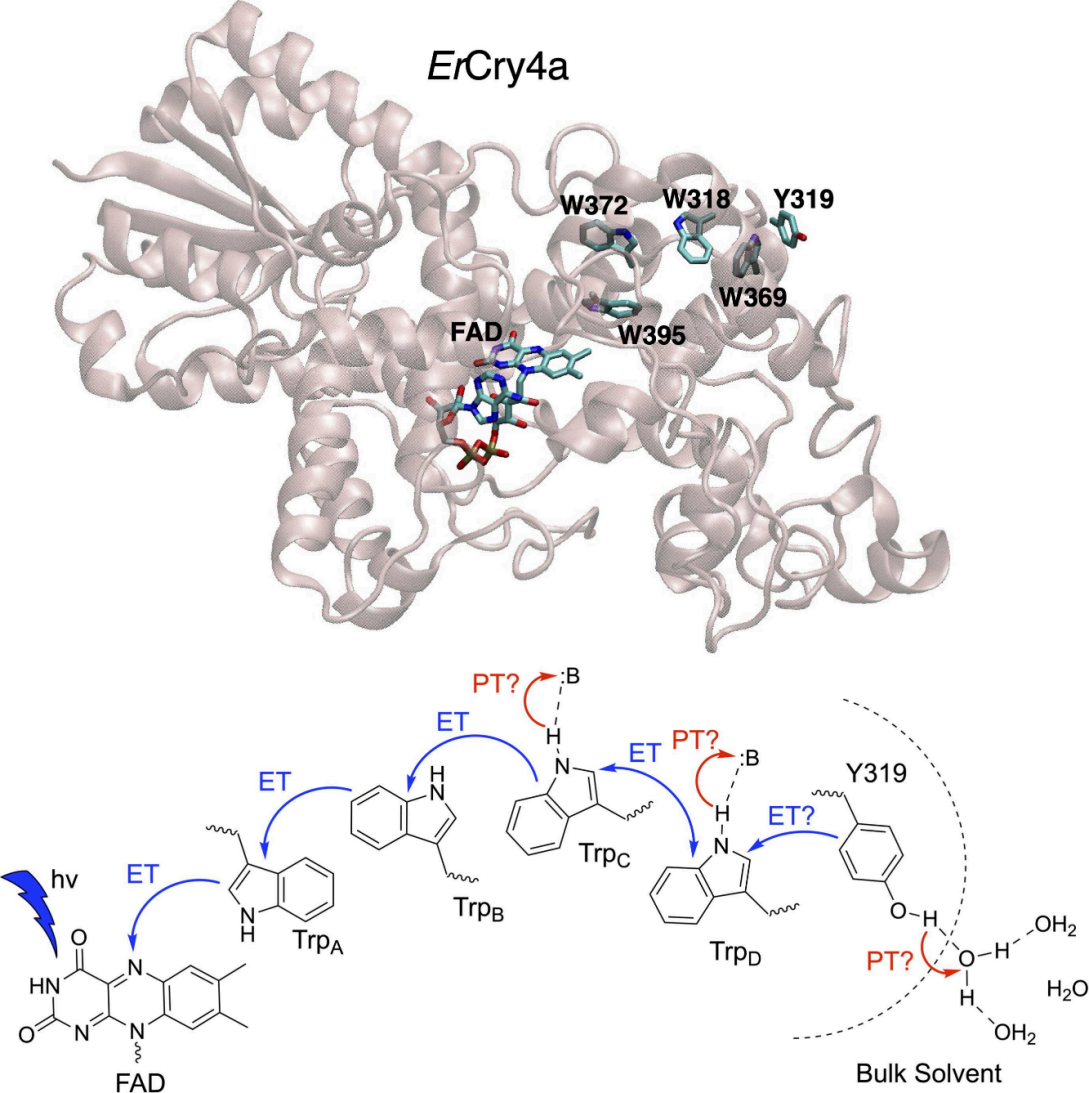
*Er*Cry4a structure (top) and the electron transfer pathway (bottom, blue arrows) along the tryptophan tetrad following blue-light excitation of the FAD chromophore. Proton transfer reactions from the third (Trp_C_) and fourth (Trp_D_) tryptophans to proton acceptors B (red arrows) are investigated herein. A possible PCET reaction between the fourth tryptophan, Trp_D_, and Y319 is also studied.

Several key gaps remain in the limited understanding of the proton-related reactions in *Er*Cry4a. In particular, it is not clear which reaction pathways occur and whether these reactions proceed at rates comparable to the radical recombination, a condition that favors magnetic sensitivity. One prominent question is the role and mechanism of a possible deprotonation of the two terminal sites, Trp_C_H^•+^ and Trp_D_H^•+^,^19,26,36^ particularly in light of recent direct transient absorption observations of Trp^•^ in chicken Cry4a.^36^ Differences in their electrostatic and hydration environments, as well as in the associated activation free energies, could significantly alter the kinetics on the μs time scale critical to magnetic sensing. Another largely unexplored question concerns the role of Y319, the tyrosine adjacent to Trp_D_.^26,35^ Tyrosine oxidation by a tryptophan radical cation, coupled to donation of the tyrosine’s phenolic proton via proton-coupled electron transfer (PCET), has precedent in the cryptochrome and photolyase families.^34,45–49^ However, its occurrence in *Er*Cry4a depends strongly on the relative positions of Trp_D_ and Y319, as well as their local environments. Given that the narrow UV–vis absorption of Tyr^•^ near 410 nm strongly overlaps with bands from various FAD and Trp species, the lack of a direct spectroscopic signature of Tyr^•^ in wild-type *Er*Cry4a does not, by itself, constitute compelling evidence against its involvement.^34,49^ Reduction of Trp_D_H^•+^ by Y319 could potentially open a viable branch in parallel with the radical recombination of RP_C_ that allows significant magnetic sensitivity.^26^

In this study, we address the two questions posed above by investigating the conformational and thermodynamic properties of the deprotonation of Trp_C_H^•+^ and Trp_D_H^•+^ and the PCET reaction involving electron transfer from Y319 to Trp_D_H^•+^ and proton transfer from Y319 to water. Starting from a structure of *Er*Cry4a modeled using AlphaFold3,^50^ classical molecular dynamics (MD) trajectories were propagated with a force field parametrized to represent the RP_C_ or RP_D_ states.^7,8^ These MD trajectories were analyzed for hydrogen-bonding interactions and water accessibility near these two tryptophan radicals. Our analysis was based on three independent 5 μs MD trajectories, corresponding to 15 μs of aggregate sampling for both RP_C_ and RP_D_, allowing extensive sampling of local hydration patterns and side chain rearrangements near the reactive sites. In addition, we used quantum mechanical/molecular mechanical (QM/MM) finite temperature string simulations with umbrella sampling to obtain free energy surfaces for the Trp_C/D_H^•+^ deprotonation and the potential PCET reaction between Trp_D_H^•+^ and Y319. The goal of these QM/MM free energy simulations was to elucidate the mechanisms and to identify the proton acceptors. The insights provided by our simulations suggest that all of these deprotonation and PCET reactions are plausible in *Er*Cry4a.

Our MD simulations show that neither Trp_C_H^•+^ nor Trp_D_H^•+^ has a nearby basic amino acid residue that could serve as an efficient proton acceptor. Previous studies reported that Trp_C_ and Trp_D_ have similar solvent exposure, with approximately five water molecules within 4 Å of each site, but did not clearly resolve hydrogen bonding at the indole nitrogen.^26,35^ Interestingly, we find here that nearly all conformations sampled along the RP_C_ trajectory feature a water molecule hydrogen bonded to the indole nitrogen of Trp_C_H^•+^. In most configurations, the hydrogen-bonded water is tightly coordinated within the protein through two additional hydrogen bonds, one to the hydroxyl oxygen of the S401 residue and the other to the backbone carbonyl oxygen of the L314 residue (Figure 2A). M307 largely blocks bulk solvent access to the indole nitrogen of Trp_C_H^•+^. Only a small fraction (3.9%) of conformations, typically those in which the hydrophobic side chain of M307 adopts an alternative dihedral angle (Figure 2B), provide a viable channel for proton transfer from Trp_C_H^•+^ to bulk solvent (Figure 2A). By contrast, Trp_D_H^•+^ has much more direct bulk solvent access. Along the RP_D_ trajectory, the indole nitrogen of Trp_D_H^•+^ retains sufficient accessibility to bulk water through a hydrogen bond in 26.9% of all sampled conformations, although it more often hydrogen bonds to the backbone oxygen of C317 (Figure 3A).

**Figure 2:**
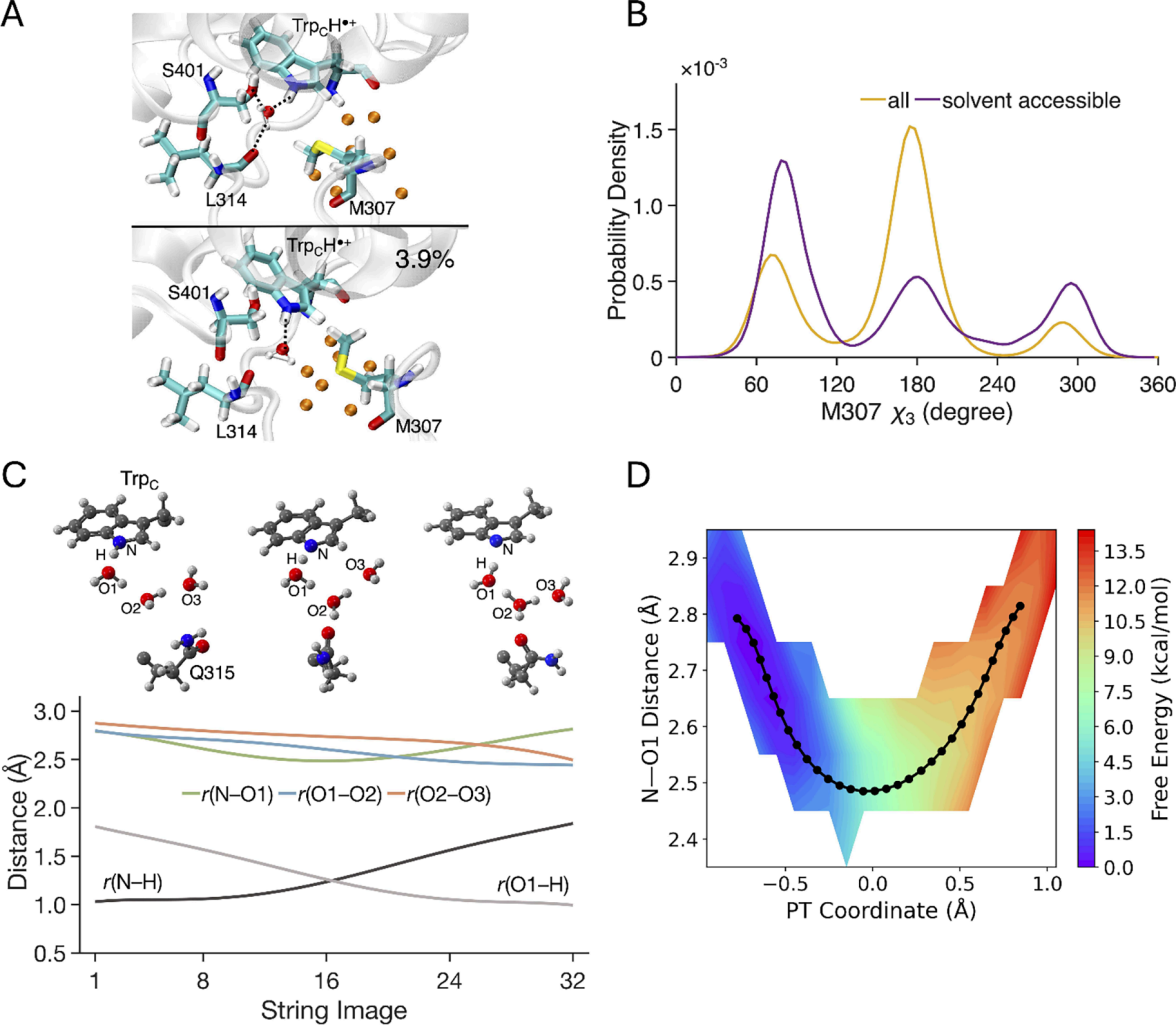
Proton transfer from Trp_C_H^•+^ to a water cluster at the protein–solvent interface. (A) A water molecule is hydrogen bonded to Trp_C_H^•+^ in all conformations sampled along the RP_C_ trajectories. In most conformations (top), the environment surrounding Trp_C_H^•+^ prevents proton release to bulk solvent. Only 3.9% of conformations (bottom) are solvent accessible, defined as exhibiting a direct hydrogen-bonding pathway connecting the water hydrogen-bonded to the indole nitrogen and bulk solvent. Bulk water molecules are shown as orange oxygen spheres. (B) Probability density of the M307 χ_3_ dihedral angle (C_β_–C_γ_–S_δ_−C_ε_) averaged over the RP_C_ trajectories. χ_3_ ≈180° is mainly associated with the closed water channel (top panel of (A)), whereas χ_3_ ≈ 75° and 285° are mainly associated with a bulk solvent accessible open water channel (bottom panel of (A)), although these trends do not always hold because other dihedral angles also impact the water channel. (C) Average reaction coordinates, defined as interatomic distances, along the converged string in the final iteration. (D) Two-dimensional free energy surface, where the PT coordinate is defined as *r*(N–H) – *r*(O1–H), and the *y*-axis is the proton donor–acceptor distance *r*(N–O1). The MFEP for the converged string is shown as the black curve.

**Figure 3:**
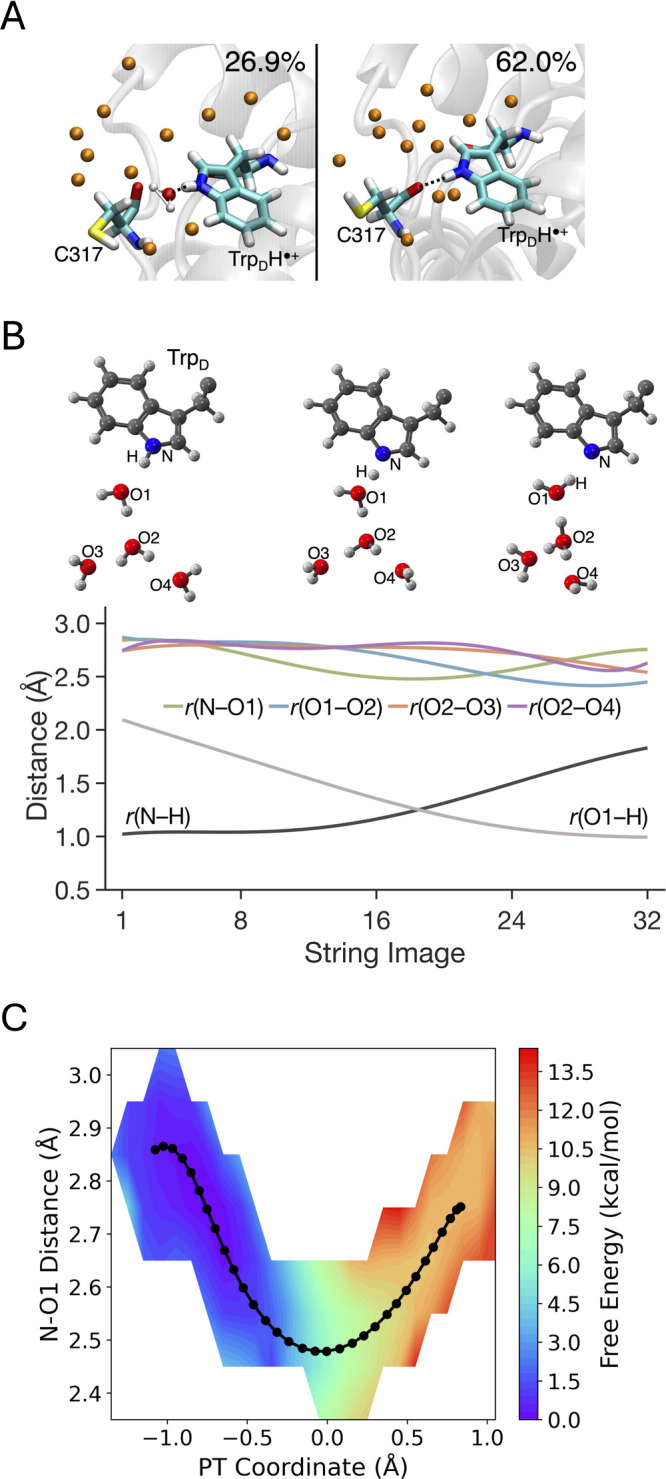
Proton transfer from Trp_D_H^•+^ to a water cluster at the protein–solvent interface. (A) For the RP_D_ trajectories, 26.9% of the conformations show the indole nitrogen hydrogen bonded to a water molecule in direct contact with bulk solvent, whereas 62.0% of the conformations show the indole nitrogen hydrogen bonded to the backbone oxygen of C317. (B) Average reaction coordinates, defined as interatomic distances, along the converged string in the final iteration. In this case, four water molecules participate in forming an Eigen cation intermediate. (C) Two-dimensional free energy surface, where the PT coordinate is defined as *r*(N–H) – *r*(O1–H), and the *y*-axis is the proton donor–acceptor distance *r*(N–O1). The MFEP for the converged string is shown as the black curve.

The primary step of tryptophan deprotonation is the transfer of the indole proton in TrpH^•+^ to a nearby preorganized water cluster that may be partially protein-bound. This local step is expected to be endergonic, given the moderate acidity of TrpH^•+^ (p*K*_a_ ≈ 4.3) compared to H_3_O^+^ (p*K*_a_ ≈ 0), corresponding to *ΔG* ^o^ ≈ 6.1 kcal/mol. However, subsequent solvent relaxation enabling proton migration into bulk solvent via the Grotthuss mechanism is expected to enable deprotonation at neutral pH. Given the complexity of simulating the entire proton transport process, our QM/MM free energy simulations focus on the local proton transfer from Trp_C/D_H^•+^ to a hydrogen-bonded water cluster. This process serves as the dominant kinetic bottleneck and depends strongly on the hydrogen-bond geometry at the indole nitrogen and the electrostatic environment at the protein–water interface.

A string simulation of the proton transfer from Trp_C_H^•+^ was initiated from a representative conformation in which the indole nitrogen is solvent accessible (lower panel of Figure 2A). The QM region consisted of Trp_C_H^•+^ and three water molecules, and five reaction coordinates were employed to describe the process. The average reaction coordinates along the minimum free energy path (MFEP) in the converged string (Figure 2C) illustrate that as the indole proton is transferred to water, a second proton transfer within the water cluster generates a hydronium that is hydrogen bonded to the carbonyl oxygen of Q315, accompanied by contraction of the interwater O–O distances to ∼ 2.5 Å. Although hydrogen-bonding interactions with Q315 may stabilize the local hydronium state, this interaction, together with the surrounding environment, may hinder further proton diffusion. The initial proton transfer process was similar for deprotonation of Trp_D_, although its greater access to bulk solvent favors an Eigen cation intermediate, H_9_O_4_^+^ (Figure 3B). In this case, the string simulation was initiated from a conformation in which the indole nitrogen is hydrogen-bonded to a water molecule (left panel of Figure 3A). The QM region consisted of Trp_D_H^•+^ and four water molecules, and six reaction coordinates were used to describe the process (Figure 3B).

The two-dimensional free energy surfaces projected onto the coordinates associated with the initial proton transfer from Trp_C/D_H^•+^ to the hydrogen-bonded water molecule, as well as the MFEP, are depicted in Figures 2D and 3C. Both free energy profiles indicate that tryptophan deprotonation is thermodynamically uphill by ∼ 10–11 kcal/mol, resulting in a plateau that corresponds to stabilization of the proton in the water cluster. Presumably, the proton would eventually diffuse to bulk water, but this diffusion cannot occur in the present simulations due to the limited QM region. Despite the similar free energy profiles, deprotonation of Trp_D_H^•+^ is expected to be more efficient than Trp_C_H^•+^, as the indole nitrogen of Trp_C_ is often surrounded by a hydrophobic cavity and thus is less accessible to bulk solvent. Specifically, the fraction of conformations that are amenable to deprotonation of TrpH^•+^ to bulk solvent is 3.9% for Trp_C_ and 26.9% for Trp_D_, leading to a greater equilibrium constant prefactor for Trp_D_. Moreover, we estimate that these conformations have a lifetime on the order of 100 ps for both Trp_C_H^•+^ and Trp_D_H^•+^ (Figure S5). These lifetimes are sufficient to enable proton diffusion to bulk solvent.

We next examine the potential multisite, orthogonal PCET involving Trp_D_ and Y319. Our MD simulations reveal that upon oxidation of Trp_D_, the side chain of Y319 adopts two main configurations: a roughly stacked arrangement with Trp_D_H^•+^ and a flipped-out orientation toward the solvent (Figure 4A). These conformations can be distinguished using the side chain dihedral angle χ_1_ of Y319. The potential of mean force (PMF) along the Y319 χ_1_ angle, computed with umbrella sampling using a molecular mechanical force field, indicates that the stacked conformation is slightly more thermodynamically favorable, and the barrier between the two conformations is ∼ 4.6 kcal/mol (Figure 4B). The stacked conformation is presumably more conducive to PCET, as it places the Y319 and Trp_D_H^•+^ rings closer to each other, decreasing the distance across which the electron must transfer. Furthermore, the PMF calculated for the Tyr^•^ state produced by the PCET reaction indicates that the Tyr^•^ is even more likely to remain stacked with Trp_D_ (Figure 4B).

**Figure 4:**
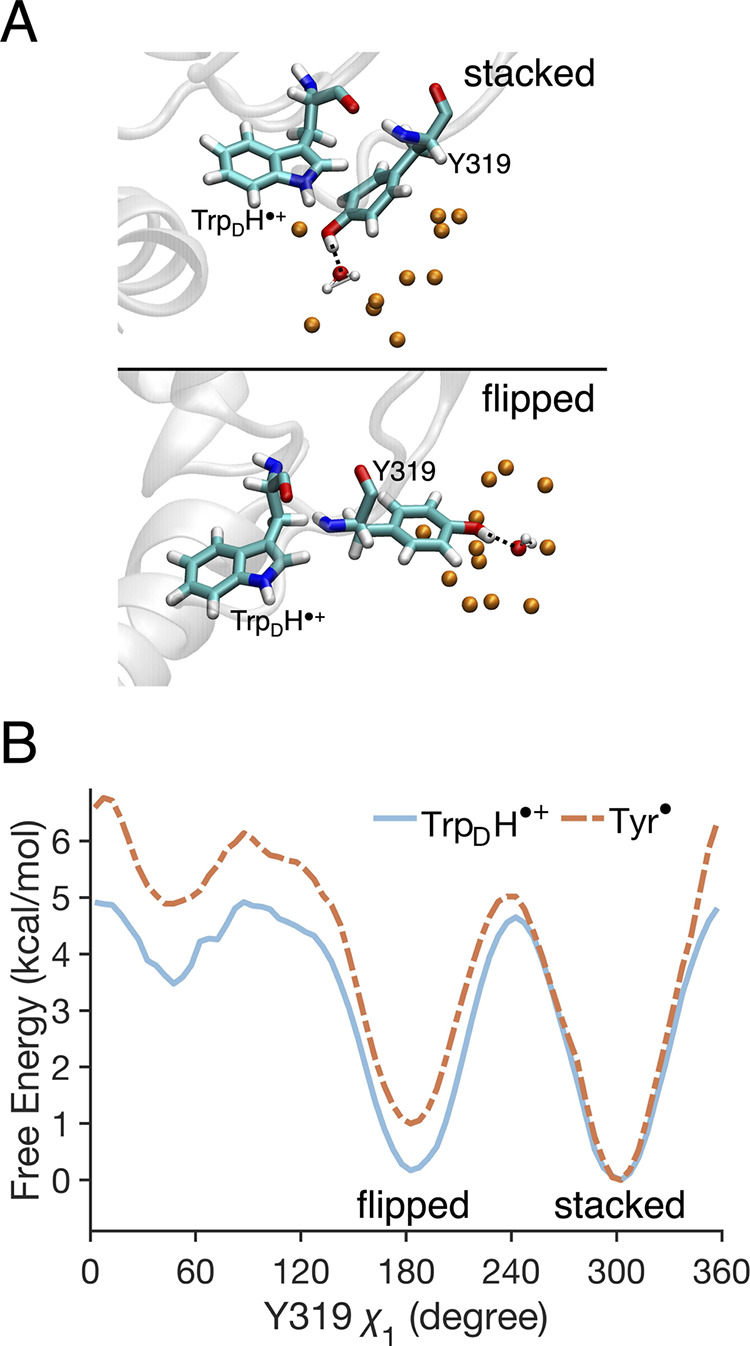
Conformational analysis of Trp_D_H^•+^ and Y319. (A) Two representative conformations along the RP_D_ trajectories, showing the stacked (top) and flipped (bottom) Trp_D_H^•+^–Y319 arrangements. (B) Potential of mean force (PMF) along the Y319 χ_1_ dihedral angle (N–C_α_–C_β_–C_γ_), obtained from umbrella sampling with a molecular mechanical force field. Here, χ_1_ ∼ 300° corresponds to the stacked conformation, and χ_1_ ∼ 180° corresponds to the flipped conformation.

A QM/MM string simulation of this PCET reaction was started from a representative stacked conformation, with the QM region composed of Trp_D_H^•+^, Y319, and four water molecules (Figure 5A). Analysis of the six reaction coordinates along the MFEP illustrates the contraction of the O–O distances within the water cluster to form an Eigen cation as proton transfer approaches completion. The two-dimensional free energy surface projected onto the coordinates associated with proton transfer from Y319 to the water molecule reveals stable minima associated with the reactant and product, connected by a free energy barrier of ∼ 9 kcal/mol for this PCET reaction (Figures 5C,D). The spin densities associated with the unpaired electron for conformations corresponding to the reactant, top of the barrier, and product (Figure 5D) clearly demonstrate concerted electron and proton movement during the radical transfer from Trp_D_ to Y319. The thermodynamically uphill *ΔG*° ≈ 7.4 kcal/mol reflects the reaction free energy of the local PCET at the protein–solvent interface in the absence of proton transport to bulk solvent, which is expected to occur but is not possible with the QM region used for these simulations. A free energy barrier of ∼ 9 kcal/mol corresponds to a sub-microsecond time scale at the physiological temperature using transition state theory (see discussion in the SI). This result suggests that PCET between Trp_D_ and Y319 is viable in *Er*Cry4a and falls within a regime where kinetic competition with radical recombination is plausible. Nevertheless, a quantitative rate theory treatment requires explicit consideration of nonadiabatic effects,^51–53^ beyond this transition state theory estimate. Such a treatment may be warranted if future experiments provide firmer evidence for the mechanistic relevance of this PCET reaction in *Er*Cry4a.

**Figure 5:**
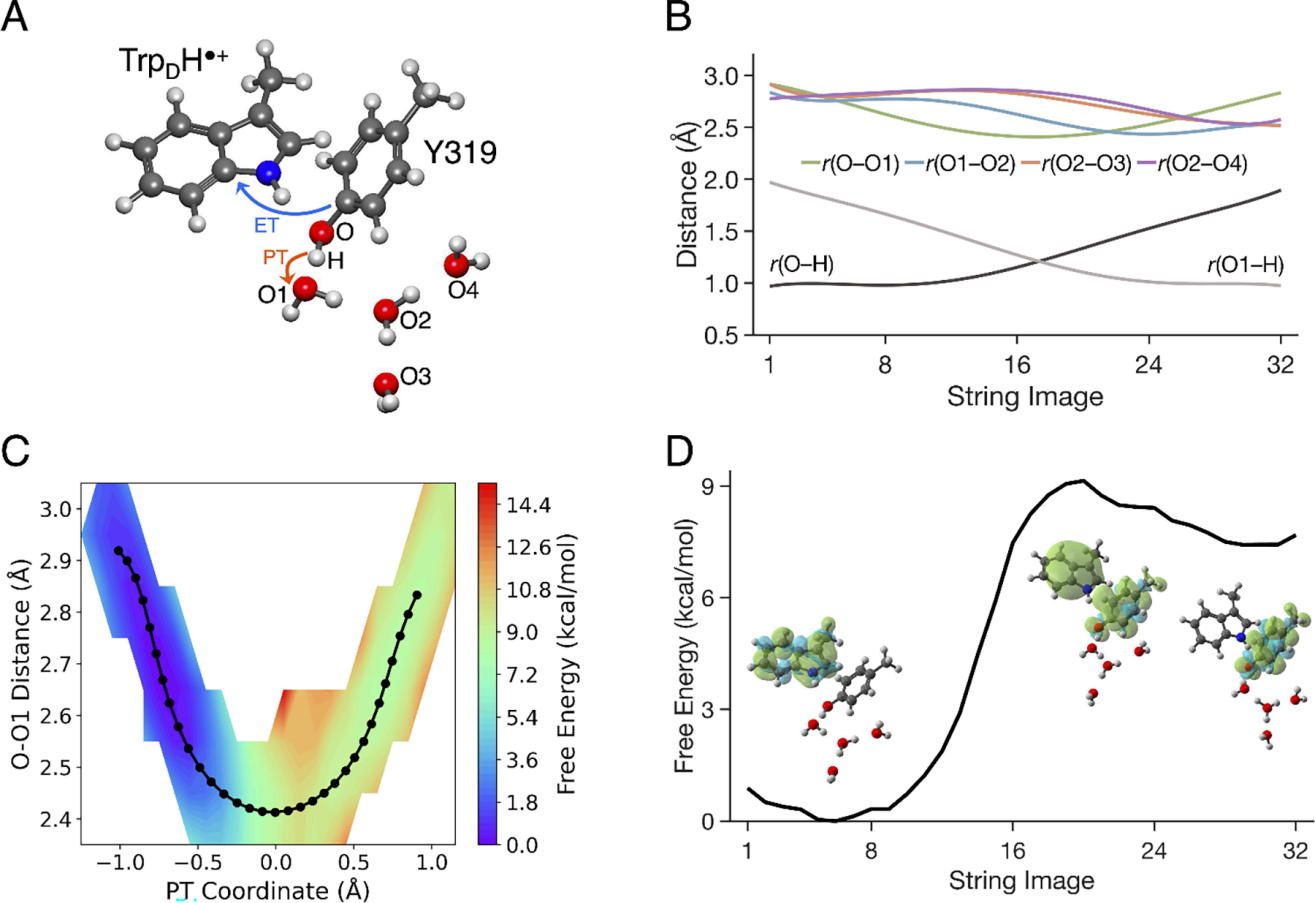
PCET between Trp_D_H^•+^ and Y319. (A) QM region with arrows indicating the ET and PT pathways. (B) Average reaction coordinates, defined as interatomic distances, along the converged string in the final iteration. (C) Two-dimensional free energy surface, where the PT coordinate is defined as *r*(O–H) – *r*(O1–H) and the *y*-axis is the proton donor–acceptor distance *r*(O–O1). The MFEP for the converged string is shown as the black curve. (D) Free energy profile along the MFEP, with spin densities for representative conformations of the reactant, where the radical is on Trp_D_H^•+^ and the proton is on Y319; the top of the barrier, where the radical is delocalized over Trp_D_H and Y319 and the proton is midway between the Y319 oxygen and water oxygen O1; and the product, where the radical is on Y319 and the proton is fully transferred from Y319, forming an Eigen cation.

In summary, this work provides mechanistic insights into the proton-related reactions for *Er*Cry4a that are closely linked to the proposed radical-pair magnetoreception. Our simulations suggest a more efficient deprotonation of Trp_D_H^•+^ relative to Trp_C_H^•+^, arising primarily from the restricted bulk solvent access to the indole nitrogen of Trp_C_, rather than from differences in the thermodynamics or kinetics of the local proton transfer reaction. Thus, in wild-type *Er*Cry4a, deprotonation of Trp_D_H^•+^ is likely to provide a more accessible reaction pathway than deprotonation of Trp_C_H^•+^ to compete with RP_C_ recombination. This finding provides support for the composite radical-pair hypothesis in which both Trp_C_H^•+^ and Trp_D_H^•+^ contribute to cryptochrome magnetoreception. Importantly, slower deprotonation could enhance magnetic field effects by competing kinetically with radical recombination while also allowing the radical pair to persist longer, potentially explaining the stronger magnetic sensitivity reported for a Trp_D_ mutant, in which RP_C_ is the terminal radical pair.^19^ Clarification of the role of Trp_C/D_H^•+^ deprotonation requires further targeted experiments. For example, mutation of M307 to a shorter, hydrophilic amino acid residue to increase solvent exposure of the indole nitrogen of Trp_C_ could alter the relative deprotonation rates of the two tryptophans and therefore influence the magnetic field effects.

Our results also show that Y319, which is at the protein surface and can adopt a nearly stacked configuration with Trp_D_, provides a feasible pathway for reduction of Trp_D_H^•+^ coupled to proton transfer from Y319 to the solvent, on an estimated time scale relevant to magnetoreception. This PCET reaction, leading to a flavin-tyrosyl radical pair that is less likely to undergo rapid recombination, introduces another possible competing pathway that could impact spin dynamics influencing magnetic field effects. Tyr^•^ could facilitate protein–protein interaction through local conformational changes in the early stages of the signal transduction cascade. Lastly, the potential role of Y319 in both magnetic sensing and signaling processes may distinguish the night-migratory songbird from nonmigratory species such as fruit flies, whose cryptochromes contain the tryptophan tetrad but lack the terminal tyrosine.^54^ Further investigation of these hypotheses requires experimental evidence for the involvement of Y319 in the photocycle. For example, similar to previous experiments on pigeon Cry4a,^34^ time-resolved spectroscopy targeting Tyr^•^ radical formation in *Er*Cry4a could provide additional insights. The theoretical work presented herein provides strong motivation for these types of experimental studies aimed at understanding magnetoreception in migratory birds.

## Supplementary Material





## Data Availability

Force field parameters and initial and final coordinates in classical MD simulations, solvent-access analysis files, AMBER/Q-Chem interface files of the QM/MM string simulations, and umbrella sampling simulation data are available in a Zenodo repository (DOI: 10.5281/zenodo.20054487).

## Notes

Computational details for obtaining partial charges for neutral tryptophan and tyrosine radicals, classical MD simulations, classical umbrella sampling, and QM/MM free energy simulations (PDF)
Representative structure files for the three protein states (RP_C_, RP_D_, and Tyr^•^) and a sample NAMD configuration file (ZIP)
