# Supplementary material for: Protein–Solvent Interface Controls Proton-Coupled Reactivity in Cryptochrome 4a
Source: J Am Chem Soc. 2026 Jul 10;148(28):29641–8. doi: 10.1021/jacs.6c09345 (PMC13397564; doi:10.1021/jacs.6c09345)
Supplement: Supplementary file 1 [file ja6c09345_si_001.pdf]

Supporting Information

Protein–Solvent Interface Controls Proton-Coupled Reactivity in Cryptochrome 4a

Jiate Luo[1], Matthew Tremblay[1], Jonathan Hungerland[2], Ilia A. Solov'yov[*,2,3,4], Joseph E. Subotnik[*,1], and Sharon Hammes-Schiffer[*,1]

[1]Department of Chemistry, Princeton University, Princeton, New Jersey 08544, United States
[2]Institut für Physik, Carl von Ossietzky-Universität Oldenburg, Carl-von-Ossietzky Str. 9-11, Oldenburg D-26129, Germany
[3]Research Center for Neurosensory, Science, Carl von Ossietzky Universität Oldenburg, 26111 Oldenburg, Germany
[4]Center for Nanoscale Dynamics (CENAD), Institute of Physics, Carl von Ossietzky Universität Oldenburg, 26129 Oldenburg, Germany

[*]E-mail: shs566@princeton.edu; subotnik@princeton.edu; ilia.solovyov@uni-oldenburg.de

Table of Contents

Partial Charges for Neutral Tryptophan and Tyrosine Radicals S2

Classical MD Simulations S4

Classical Umbrella Sampling Simulations S8

QM/MM Free Enegry Simulations S10

References S16

Partial Charges for Neutral Tryptophan and Tyrosine Radicals

Neutral tyrosyl and tryptophanyl radical residues were generated by removing the phenolic hydrogen of tyrosine or the indole hydrogen of tryptophan, respectively, giving systems with total charge 0 and spin multiplicity 2. Each residue was capped with N-acetyl and N-methylamide groups to preserve the local backbone electrostatic environment used in the CHARMM36m fixed-charge framework. Initial capped-residue structures were energy-minimized with the GAFF force field using the conjugate-gradient algorithm to provide physically reasonable starting geometries. The resulting structures were then optimized at the unrestricted r^2SCAN-3c level of theory using ORCA 6.0, with the default integration grid and tight self-consistent field (SCF) convergence criteria.

Electrostatic potentials of the neutral tryptophan and tyrosine radicals were calculated at the HF/6-31G(d) level on the r^2SCAN-3c-optimized geometries. Restrained electrostatic potential (RESP) fitting was performed using the built-in ORCA implementation with a hyperbolic penalty function. During the fitting procedure, atomic charges on the backbone and capping groups were fixed to their canonical CHARMM36m values by assigning reference charges with very large restraint weights. Side-chain charges were initialized from the corresponding CHARMM36m parameters and optimized against the quantum mechanical electrostatic potential while preserving the total residue charge. Charges on chemically equivalent atoms were symmetrized by averaging the fitted values. All quantum mechanical calculations were performed as unrestricted open-shell calculations with total charge 0 and spin multiplicity 2. The resulting RESP charges are given in Tables S1 and S2 and reproduce the electrostatic potential of the neutral radical residues while maintaining compatibility with the CHARMM36m fixed-charge force-field framework.

Table S1. Partial Charges and Atom Types of Tyr$^{\bullet}$ Used in the Simulations.

Atom name	Atom type	Charge	Atom name	Atom type	Charge
CB	CT2	-0.1746	HE1	HP	0.1557
HB1	HA2	0.0664	CZ	CA	0.6446
HB2	HA2	0.0664	OH	OH1	-0.4606
CG	CA	0.1916	CD2	CA	0.2142
CD1	CA	-0.2142	HD2	HP	0.1643
HD1	HP	0.1643	CE2	CA	-0.2727
CE1	CA	-0.2727	HE2	HP	0.1557

Table S2. Partial Charges and Atom Types of Trp$^{\bullet}$ Used in the Simulations.

Atom name	Atom type	Charge	Atom name	Atom type	Charge
CB	CT2	-0.1642	CE3	CAI	-0.1396
HB1	HA2	0.1091	HE3	HP	0.1480
HB2	HA2	0.1091	CZ3	CA	-0.2233
CG	CY	-0.1039	HZ3	HP	0.1637
CD1	CA	0.2765	CZ2	CAI	-0.3545
HD1	HP	0.0944	HZ2	HP	0.1892
NE1	NY	-0.6557	CH2	CA	-0.1101
CE2	CPT	0.5997	HH2	HP	0.1523
CD2	CPT	-0.0907			

Classical MD Simulations

The initial protein structure was predicted for the complete *European robin* cryptochrome 4a (*Er*Cry4a) sequence[2] using the top ranked model of AlphaFold3.[3] The default AlphaFold3 (AF3) settings were used for structure prediction with a single seed (2099934013). FAD was incorporated through co-folding with the protein. As expected, the overall fold of the predicted structure (see Fig. S1A) is similar to previously reported homology models,[1] with the main differences occuring in flexible regions including the phosphate binding loop and the C-terminal.

As shown in Figure S1B, we compared different structural models of *Er*Cry4 and *Cl*Cry4 (pigeon cryptochrome 4) using backbone root mean square deviations (RMSDs).

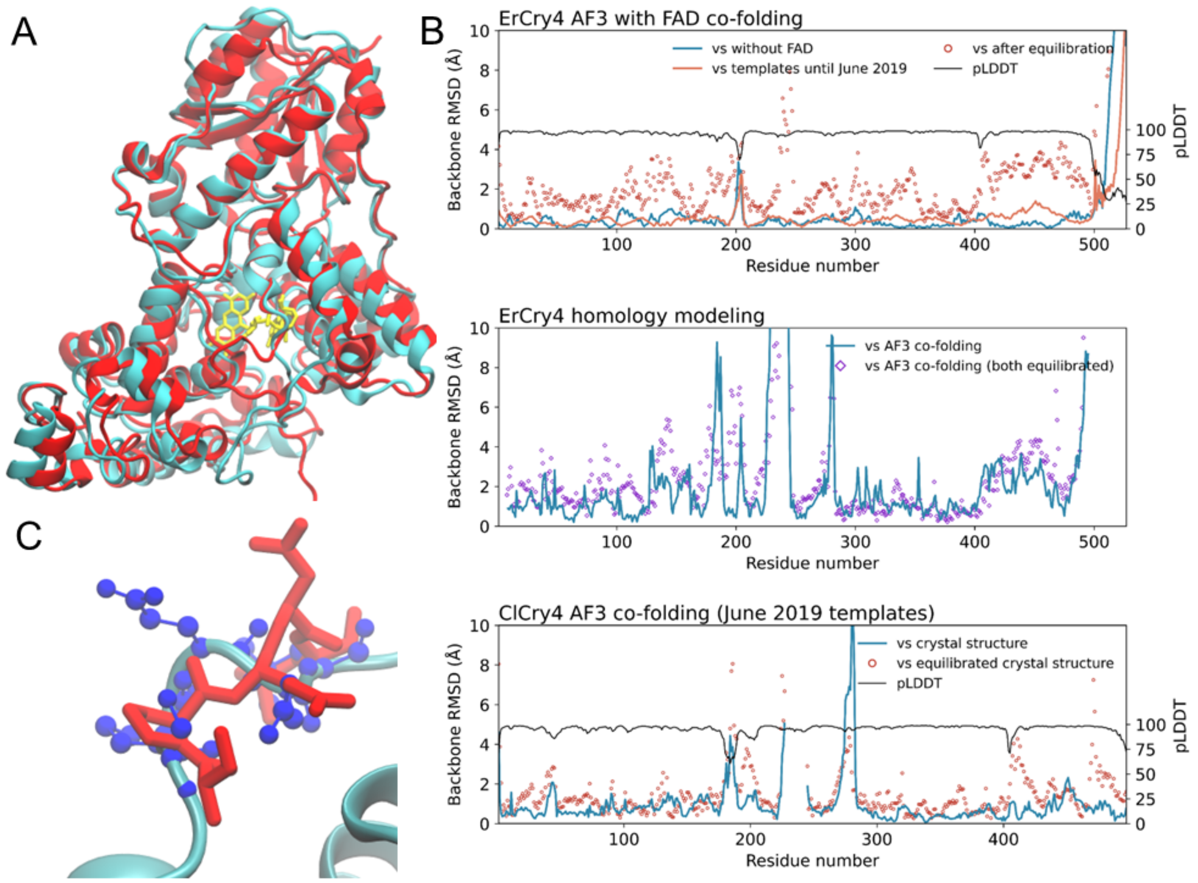

Figure S1: Structure prediction benchmarks. (A) Overall fold of the AlphaFold3 (AF3) model (red) and the previously studied homology model (cyan) of *Er*Cry4.[1] The flavin adenine dinucleotide (FAD) is shown in yellow. (B) Backbone RMSDs between different structural models of *Er*Cry4 (European robin) and *Cl*Cry4 (pigeon). The predicted Local Distance Difference Test (pLDDT) score is a confidence estimate by the AF3 model. In the lowest panel, only templates until June 2019 were included in the prediction. (C) Comparison of residues 200-204 in AF3 co-folding with FAD (blue ball-and-stick) and AF3 prediction without FAD (red licorice).

Co-folding with FAD primarily affected the C-terminus region and a proline-containing loop near residue 202, with the corresponding atomistic conformations shown in Fig. S1C. Excluding templates deposited after June 2019, including the pigeon Cry4 crystal structure 6PUO,[4] had only a minor effect on the predicted fold. By comparison, structural changes arising during MD equilibration were substantially larger. The previously used homology model predicts a somewhat different structure, with notable deviations around residues 180, 200, 220, and 280. Importantly, however, the regions surrounding the FAD and tryptophan chain remain structurally conserved. To further benchmark the AF3 approach, we predicted the *Cl*Cry4 structure while excluding templates after June 2019. The resulting model closely reproduces the experimental crystal structure except for flexible regions around residues 180, 220, and 280. Similar deviations are also observed when comparing the predicted structure with equilibrated MD structures derived from the crystal structure reported by Schuhmann *et al.*[5]

We note, however, that AF3 may still contain learned information from the pigeon Cry4 crystal structure. Excluding templates reduces direct template bias in the *Cl*Cry4 prediction, but it does not guarantee that the crystal structure was absent from the AF3 training data. Strict independence would be ensured only if *Cl*Cry4 had been part of the validation or test sets, which cannot be verified because the relevant training details are proprietary. Therefore, the strong agreement between AF3 and the *Cl*Cry4 crystal structure should be interpreted with some caution.

The structure was initially prepared in the so-called *dark state*, with the flavin part of the flavin adenine dinucleotide (FAD) and all tryptophans in a charge neutral and closed-shell state. Protonation state evaluation via PROPKA3[6] suggested protonation of GLU104. The protein structure was solvated in a cubic box of 140 Å side length to avoid potential artifacts in case of unfolding of the C-terminal. Neutralization and ionization of the solvent was achieved via the placement of sodium and chloride ions to mimic a salt concentration of 0.15 mol/L. Hydrogen mass repartitioning[7] was utilized. System preparation was performed in VMD.[8]

The CHARMM36m protein force field[9–11] was employed for the protein and solvent. The negatively charged flavin radical state was described by parameters from Aleksandrov,[12] who refined the earlier parametrization,[13,14] while the positive tryptophan radical state was described by revised partial charge parameters without modifying atom types or valence parameters.[13,14]

Molecular dynamics simulations were performed with NAMD3.[15,16] The following sim-

ulation parameters remained identical across different stages. Nonbonded interactions were excluded following a 1-3 scheme with 1-4 interaction scaling set to 1. The nonbonded cutoff radius of 12 Å was linearly introduced starting at t he switch distance of 10 Å. Nonbonded interactions were calculated at every step while the full long-range electrostatics were computed every second step. Long-range electrostatics were approximated by particle mesh Ewald summation at 1.0 Å grid-spacing.[17] To allow retention of the large outer timestep for full electrostatics even at a 4 fs inner timestep, the more stable c2 interpolation was used.[18,19] Hydrogen bonds for water molecules were restrained via the analytical SETTLE algorithm,[20] while all other hydrogen bonds were restrained by the numerical SHAKE algorithm.[21] The temperature was controlled at 300 K via a Langevin thermostat acting on all non-hydrogen atoms with a damping constant of 5 ps^{-1}. Pressure control, where active, used the Langevin piston algorithm[22,23] set at 1 atm pressure with a piston period of 200 ps and a decay constant of 50 ps. Pressure acted isotropically on all simulation box sides.

The simulation was initiated with 20000 conjugate gradient minimization steps. A 10 ns long pressure equilibration using a 1 fs simulation time step allowed the box size to adjust to the equilibrium solvent density. A 1 μs simulation in the NVT ensemble and using a 4 fs integration timestep followed. The *Er*Cry4a structure was then changed to resemble the RP_C or RP_D redox state by modifying the topology of the flavin and the protein, accordingly. The topology modifications preserved the atom order such that the exact same set of coordinates and velocities from the binary restart files were used to continue the simulation without introducing artifacts due to otherwise necessary additional minimizations or velocity re-initializations. Three replica simulations were performed for 5 μs in the RP_C and RP_D states, totaling 15 μs of data per considered radical pair state. Figure S2 shows root-mean-square-deviation and root-mean-square-fluctuation analysis for those trajectories.

To enable classical molecular dynamics simulations of the tyrosine neutral radical state of *Er*Cry4a, we selected a representative conformation with favorable water coordinations from the RP_D trajectories. The tryptophan was returned to its neutral closed shell form, and the tyrosine (Y319) was assigned a new set of partial charges, summarized in Table S1. To preserve charge neutrality, an ion was deleted that was visually determined to be sufficiently far apart from any periodic image of the protein in order to cause minimal disturbance. Three replicas were simulated that employed the same simulation settings as described. However, after the pressure equilibrations, only 250 ns of dynamics with a

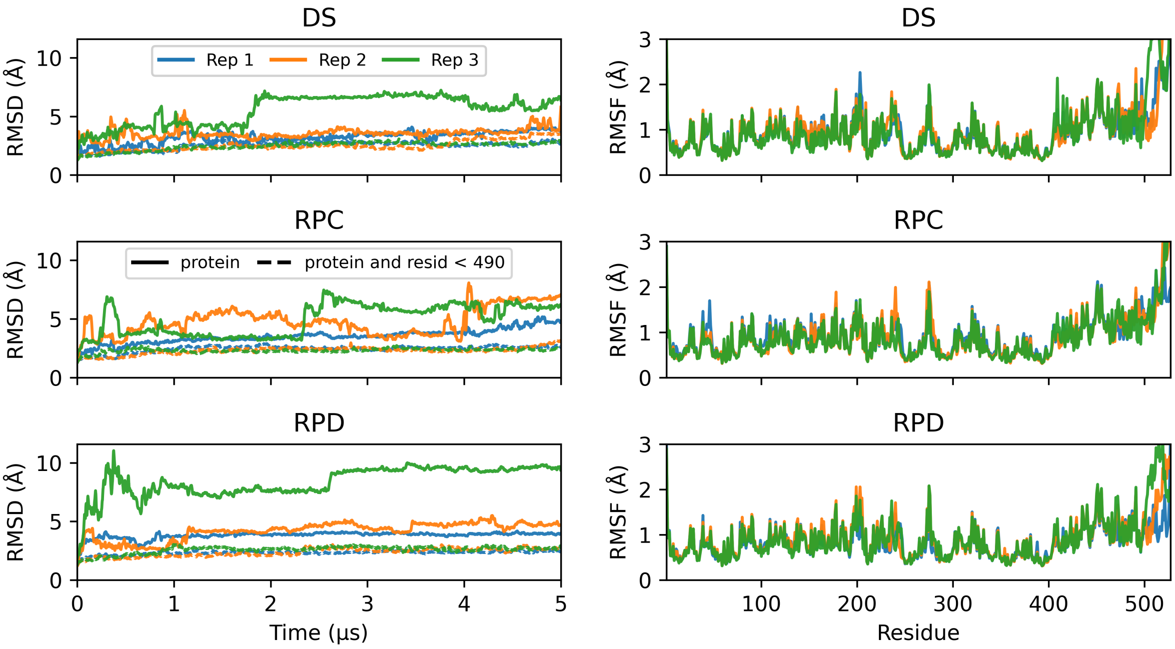

Figure S2: Root-mean-square-deviation (RMSD) and root-mean-square-fluctuation (RMSF) of the simulated trajectories. (Left) RMSD of the dark state (DS) and radical pair states (RPC, RPD) for different selections of the protein. A running average over a 10 ns window is shown. (Right) RMSF of non-hydrogen atoms of different protein residues. Fluctuations were normalized to represent the average fluctuation expected over a 10 ns time window.

2 fs simulation timestep were carried out.

Classical Umbrella Sampling Simulations

Umbrella sampling[24] was used to compute the potential of mean force (PMF) with respect to the χ_1 sidechain dihedral angle (N–C_α–C_β–C_γ) of Y319 in *Er*Cry4a. Two simulations were performed, one assuming *Er*Cry4a to have TrpD in the $\mathrm{Trp_DH^{\bullet+}}$ ($\mathrm{RP_D}$) state and one with Y319 in the $\mathrm{Tyr^{\bullet}}$ state. In the initial structure of *Er*Cry4a chosen for the $\mathrm{Trp_DH^{\bullet+}}$ simulation, Y319 was in the stacked conformation with its χ_1 angle around 305°. In the case of the $\mathrm{Tyr^{\bullet}}$ state simulation of *Er*Cry4a, the Y319 radical had a dihedral angle of $\chi_1 \sim 315°$ in the chosen initial protein structure. For both systems, 72 windows were used for the umbrella sampling. In each window, the structure was equilibrated with harmonic restraints on χ_1 centered at values ranging from 0° to 355° in intervals of 5°. The final geometry from the equilibration step of each umbrella sampling window was used to start a production trajectory with the same harmonic restraint, and the value of χ_1 was recorded every tenth time step.

The weighted histogram analysis method (WHAM) was used to compute the PMF from the recorded values of χ_1. To confirm the convergence of the simulations, PMFs were computed separately for the first and second halves of the production trajectories (Figure S3). The curves corresponding to the first and second halves of the data set agree well with one another and with the PMF obtained from the full data set.

These simulations were performed with Amber24.[25] The classical force field parameters used in all simulations were the same as those used for the unbiased MD simulations. The NVT statistical ensemble was utilized with an integration timestep of 1 fs. The *Er*Cry4a structures with the $\mathrm{Trp_DH^{\bullet+}}$ radical were equilibrated for 20 ns, while those with the $\mathrm{Tyr^{\bullet}}$ radical were equilibrated for 40 ns to ensure adequate equilibration. All production trajectories were propagated for 20 ns. The Langevin thermostat was used to maintain a temperature of 310 K, with a damping coefficient $\gamma = 2\ ps^{-1}$. All covalent bonds containing hydrogen atoms were constrained using the SHAKE algorithm. All harmonic restraints used a force constant of $k = 200$ kcal/mol/radian^2, employing the Amber convention of $U = k(\phi - \phi_0)^2$. Alan Grossfield's implementation of the WHAM algorithm[26] was used to generate the PMFs.

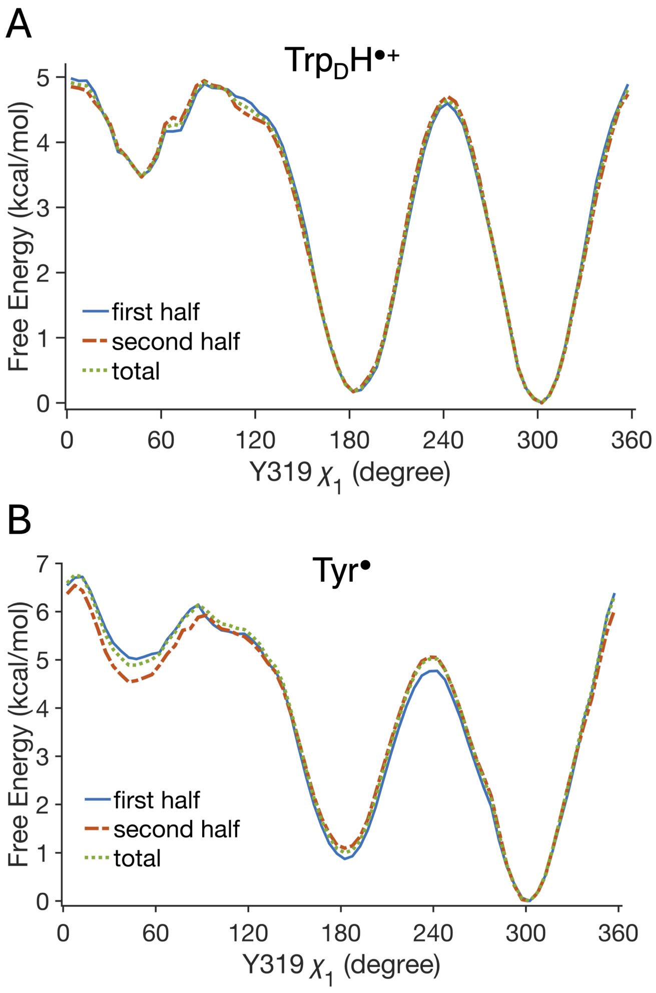

Figure S3: Convergence analysis for the classical umbrella sampling simulations for the (A) $Trp_DH^{\bullet+}$ and (B) $Tyr^{\bullet}$ states of *Er*Cry4a. The lines for each state correspond to PMFs calculated from the entire production trajectory of all umbrella sampling windows or with either the first or second half of the production trajectory. The agreement between the three lines indicates that the systems were adequately equilibrated. For comparison, the lines were aligned such that the lowest minimum corresponds to 0 kcal/mol.

QM/MM Free Energy Simulations

The QM/MM finite temperature string method with umbrella sampling was used to investigate proton transfer (PT) from $Trp_CH^{\bullet+}$ and $Trp_DH^{\bullet+}$ to water, as well as the proton-coupled electron transfer (PCET) involving $Trp_DH^{\bullet+}$ and Y319 in *Er*Cry4a. The details of this methodology are presented elsewhere,[27–29] while here we provide only a brief summary. In this approach, a string is composed of a series of images, each associated with specified values of the reaction coordinates, that connect the reactant and the product. Starting with an initial string, an iterative procedure is used to generate the minimum free energy path (MFEP) connecting the reactant and the product. Each iteration entails 100 fs of QM/MM MD sampling for each image with harmonic restraints applied to the reaction coordinates. Prior to each iteration, the average reaction coordinates from the previous set of images are used to construct an updated string by quadratic interpolation, and a new set of images is redistributed evenly along the new string. Each image is associated with the values of the reaction coordinates determined from this procedure, and these values are used to define the harmonic restraints in the QM/MM MD sampling. The reaction coordinate values associated with the harmonic restraints for each image evolve toward the MFEP. This procedure is continued iteratively until convergence.

We used the following protocol for the string simulations in this work. The restraint force constants were set to 100 kcal/(mol·Å^2) in most cases, but for images that were more challenging to sample, particularly those where the proton was shared between the indole nitrogen (or phenolic oxygen) and the water oxygen, stronger restraints of 200 to 400 kcal/(mol·Å^2) were applied. A string was considered to be converged when the changes in the reaction free energy and free energy barrier over the last five iterations remained within 1.0 kcal/mol (Figure S4). Data from all the QM/MM string iterations were used to generate the multidimensional free energy surface using the binless weighted histogram analysis method (WHAM).[26] The bin size used for plotting was set to 0.1 Å, and the convergence criterion of 0.001 was used for the WHAM procedure. The last iteration of the converged string was used to generate the MFEP.

All simulations were carried out with the Amber/Q-Chem interface.[30] Link atoms were introduced automatically by Amber at the Cα–Cβ bond across the QM/MM boundary. The QM region was treated with the range-separated hybrid functional ωB97X-D and the 6-31+G** basis set, while the MM region was described with the CHARMM36m force field and TIP3P water. This level of theory was found previously to be suitable for modeling

PCET reactions between tyrosine residues by comparison to complete active space self-consistent field (CASSCF) with perturbation theory (NEVPT2) calculations for proton potential energy profiles.[29] The QM water clusters used in the present calculations were chosen to include the local water network required to describe the initial proton-transfer process and proton stabilization in water. Simulation of the complete proton migration to bulk solvent, including back proton transfer and escape probabilities, is beyond the scope of this work. The SCF convergence criterion in the DFT calculations was set to 10^{-6} Hartree. Each independent string was started from an MD equilibrated solvated protein system. Due to the computational limitations in constructing the non-bonded pairwise interaction list, all water molecules and ions beyond 8 Å of any amino acid residues or the FAD cofactor were removed from the system unless they were within 36 Å of the indole nitrogen of $\mathrm{Trp_CH^{\bullet+}}$ or $\mathrm{Trp_DH^{\bullet+}}$. All nonbonded interactions were included without a cutoff. For each string simulation, a large mobile MM region was defined, and all other MM atoms were frozen during the simulation to maintain the integrity of the solvated protein system.

Proton transfer from $\mathrm{Trp_CH^{\bullet+}}$ to water

For this string simulation, the QM region included the side chain of the $\mathrm{Trp_CH^{\bullet+}}$ residue of *Er*Cry4a, the water molecule hydrogen bonded to the indole nitrogen of $\mathrm{Trp_CH^{\bullet+}}$ (WAT1), and two additional water molecules in the hydrogen-bonded cluster (i.e., WAT2, which is hydrogen bonded to WAT1, and WAT3, which is hydrogen bonded to WAT2 and exposed to bulk solvent). Five reaction coordinates were used to describe the proton transfer process, defined by distances involving the indole nitrogen of $\mathrm{Trp_CH^{\bullet+}}$ (N), the transferring proton (H^+), and the oxygen atoms of the three waters (O1, O2, and O3): r(N–H), r(O1–H), r(N–O1), r(O1–O2), and r(O2–O3). Initial geometries corresponding to the reactant, product, and top of the barrier states were obtained by placing the proton next to the N atom of $\mathrm{Trp_CH^{\bullet+}}$, next to the O1 atom of WAT1, and at the midpoint between the N and O1 atoms, respectively, followed by 120 steps of energy minimization of the QM region atoms with harmonic restraints using force constants of 200 kcal/(mol·Å^2) on the reaction coordinates. During the minimization of the product state, an additional restraint was applied to the distance between the O2 atom and the proton located between the O1 and O2 atoms to localize this proton next to the O2 atom and thereby form a hydronium ion. The positions of the atoms in the MM region were fixed during these energy minimizations. An initial string composed of 32 images was generated using

quadratic interpolation connecting the reaction coordinates associated with the atoms of the QM region for these three partially optimized initial structures. Then a 1.2-ns MD trajectory equilibrating the mobile MM region, which included all atoms outside the QM region but within 22 Å of the $Trp_CH^{\bullet+}$ indole nitrogen, was propagated for each image. For this MD equilibration, the partial charges of the QM-region atoms were linearly interpolated between the first image, corresponding to the $Trp_CH^{\bullet+} + WAT1 + WAT2 + WAT3$ state of the system, and the last image, corresponding to $Trp_C^{\bullet} + WAT1 + H_3O^+ + WAT3$. The partial charges employed for the $Trp_C^{\bullet}$ radical in the charge interpolation are given in Table S2. Lastly, a 400-fs QM/MM MD equilibration of both the QM and the MM mobile region was performed with 200 kcal/(mol·Å^2) harmonic restraints on the reaction coordinates for each image.

Proton transfer from $Trp_DH^{\bullet+}$ to water

In this case, the QM region included the side chain of the $Trp_DH^{\bullet+}$ residue of *Er*Cry4a, the water molecule (WAT1) hydrogen bonded to the indole nitrogen, and three additional water molecules (WAT2, WAT3, and WAT4) in the hydrogen-bonded water cluster. Six reaction coordinates were used to describe the process, defined by the distances involving the indole nitrogen of the $Trp_DH^{\bullet+}$ atom (N), the transferring proton (H^+), and the oxygen atoms of the four water molecules (O1, O2, O3, and O4): r(N–H), r(O1–H), r(N–O1), r(O1–O2), r(O2–O3), and r(O2–O4). Initial geometries of the system for the reactant, product, and top of the barrier were obtained by placing the proton next to the N atom of $Trp_DH^{\bullet+}$, next to the O1 atom of WAT1, and at the midpoint between the N and O1 atoms, respectively, followed by 120 steps of energy minimization for the QM region atoms with harmonic restraints using force constants of 200 kcal/(mol·Å^2) on the reaction coordinates. During the minimization of the product state, an additional restraint was applied to the distance between the O2 atom and the proton located between the O1 and O2 atoms to localize this proton next to the O2 atom and thereby form a hydronium ion. The positions of the atoms in the MM region were fixed during these energy minimizations. An initial string composed of 32 images was generated using quadratic interpolation connecting the reaction coordinates associated with the QM region atoms for these three partially optimized initial structures. Then a 1.2-ns MD trajectory equilibrating the mobile MM region, which included all atoms outside the QM region but within 22 Å of the $Trp_DH^{\bullet+}$ indole nitrogen, was propagated for each image. For this MD equilibration, the partial charges of the QM-region atoms were linearly interpolated

between the first image, corresponding to the $Trp_DH^{\bullet+}$ + WAT1 + WAT2 + WAT3 + WAT4 state, and the last image, corresponding to $Trp_D^{\bullet}$ + WAT1 + H_3O^+ + WAT3 + WAT4. The partial charges employed for the $Trp_D^{\bullet}$ radical in the charge interpolation are given in Table S2. Lastly, a 400-fs QM/MM MD equilibration of both the QM and the MM mobile region was performed with 200 kcal/(mol·Å^2) harmonic restraints on the reaction coordinates for each image.

Proton coupled electron transfer (PCET) between $Trp_DH^{\bullet+}$ and Y319

For this string simulation, the QM region included the side chains of the $Trp_DH^{\bullet+}$ and Y319 residues of *Er*Cry4a, the water molecule (WAT1) hydrogen bonded to the phenolic oxygen, and three additional water molecules (WAT2, WAT3, and WAT4) in the hydrogen-bonded water cluster. Six reaction coordinates were used to describe the PCET process, defined by distances involving the phenolic oxygen of Y319 (O), the transferring proton (H^+), and the oxygen atoms of the four water molecules (O1, O2, O3, and O4): r(O–H), r(O1–H), r(O–O1), r(O1–O2), r(O2–O3), and r(O2–O4). Initial geometries corresponding to the reactant, product, and top of the barrier states were obtained by placing the proton next to the O atom of the Y319 residue, next to the O1 atom of WAT1, and at the midpoint between the O and O1 atoms, respectively, followed by 120 steps of energy minimization of the QM region atoms with harmonic restraints using force constants of 200 kcal/(mol·Å^2) on the reaction coordinates. During the minimization of the product state, an additional restraint was applied to the distance between the O2 atom and the proton located between the O1 and O2 atoms to localize this proton next to the O2 atom and thereby form a hydronium ion. The positions of the atoms in the MM region were fixed during these energy minimizations. An initial string composed of 32 images was generated using quadratic interpolation connecting the reaction coordinates associated with the atoms of the QM region for these three partially optimized initial structures. Then a 1.2-ns MD trajectory equilibrating the mobile MM region, which included all atoms outside the QM region but within 22 Å of the O atom of the Y319 residue, was propagated. For this MD equilibration, the partial charges of the QM-region atoms were linearly interpolated between the first image, corresponding to the $Trp_DH^{\bullet+}$ + Y319 + WAT1 + WAT2 + WAT3 + WAT4 state, and the last image, corresponding to the Trp_DH + $Tyr^{\bullet}$ + WAT1 + H_3O^+ + WAT3 + WAT4 state. The partial charges employed for the $Tyr^{\bullet}$ radical in the charge interpolation are given in Table S1. Lastly, a 400-fs QM/MM MD equilibration of the QM and the MM mobile region was

performed with 200 kcal/(mol·Å^2) harmonic restraints on the reaction coordinates for each image.

The rate constant for a free energy barrier of $\sim$ 9 kcal/mol can be estimated to be 2.9×10^6 s^{-1} using transition state theory with a prefactor of $k_\mathrm{B}T/h = 6.46 \times 10^{12}$ s^{-1} at $T = 310$ K, neglecting dynamical barrier recrossings and tunneling. Given the uncertainties of the QM/MM free energy barriers, this work suggests a PCET rate constant in the approximate range of 10^5 – 10^8 s^{-1}. This range is comparable to representative radical-pair recombination timescales ranging from several hundred nanoseconds to several microseconds obtained from previous experimental and theoretical studies.[1,31–33] However, if this PCET reaction is nonadiabatic, TST is not applicable, and a vibronically nonadiabatic PCET rate constant expression must be used.[34,35] Such a calculation is beyond the scope of the present work.

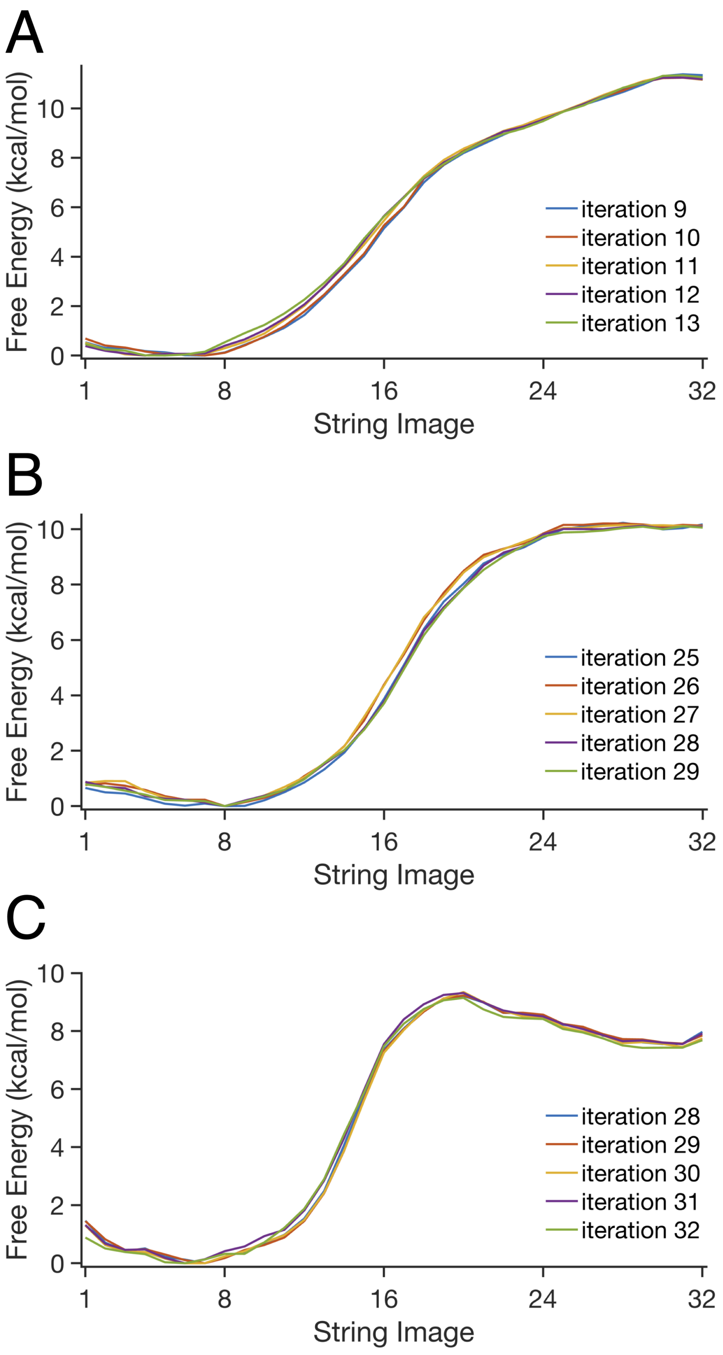

Figure S4: Free eneregy profiles computed along the MFEPs for the last five iterations. (A) PT from $\mathrm{Trp_C H^{\bullet +}}$. (B) PT from $\mathrm{Trp_D H^{\bullet +}}$. (C) PCET involving $\mathrm{Trp_D H^{\bullet +}}$ and Y319.

Analysis of Conformations Conducive to Proton Diffusion from Tryptophan Radical Cation to Bulk Water

As discussed in the main text, we estimated that the percentage of sampled conformations amenable to deprotonation of $TrpH^{\bullet+}$ to bulk solvent through a hydrogen-bonded network is 3.9% for Trp_C and 26.9% for Trp_D. We estimated the apparent lifetimes of these conformations by fitting the distribution to an exponential, leading to decay time constants of τ = 99.5 ps and 88.1 ps for Trp_CH^+ and Trp_DH^+, respectively (Figure S5). Based on this analysis, we estimate that these conformations allowing proton diffusion to bulk water have a lifetime on the order of 100 ps.

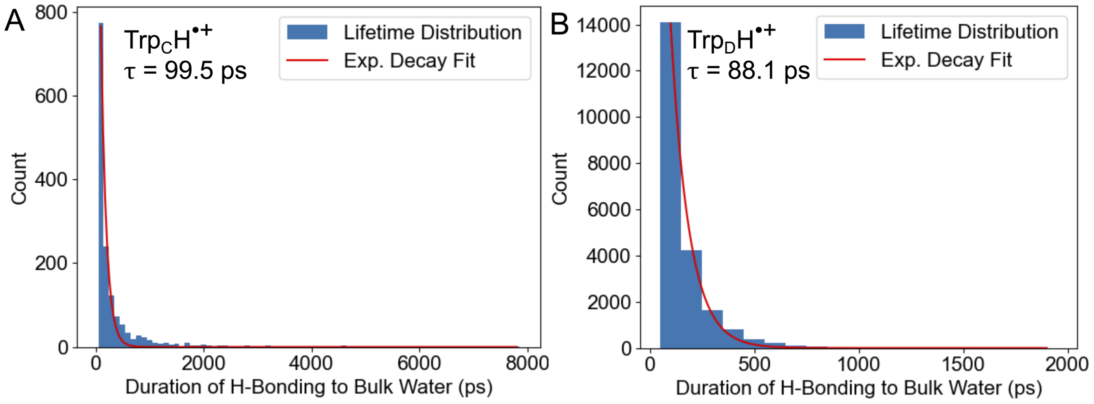

Figure S5: Distribution of the lifetimes of conformations conducive to proton diffusion from $TrpH^{\bullet+}$ to bulk water for (A) $Trp_CH^{\bullet+}$ and (B) $Trp_DH^{\bullet+}$. The histogram of the observed lifetimes is shown in blue, and an exponential fit ($f(t) = Ae^{-t/\tau}$) is shown in red. The resulting time constants are τ =99.5 ps and 88.1 ps, respectively.

References

[1] Xu, J.; Jarocha, L. E.; Zollitsch, T.; Konowalczyk, M.; Henbest, K. B.; Richert, S.; Golesworthy, M. J.; Schmidt, J.; Déjean, V.; Sowood, D. J. C.; Bassetto, M.; Luo, J.; Walton, J. R.; Fleming, J.; Wei, Y.; Pitcher, T. L.; Moise, G.; Herrmann, M.; Yin, H.; Wu, H.; Bartölke, R.; Käsehagen, S. J.; Horst, S.; Dautaj, G.; Murton, P. D. F.; Gehrckens, A. S.; Chelliah, Y.; Takahashi, J. S.; Koch, K.-W.; Weber, S.; Solov'yov, I. A.; Xie, C.; Mackenzie, S. R.; Timmel, C. R.; Mouritsen, H.; Hore, P. J. Magnetic Sensitivity of Cryptochrome 4 from a Migratory Songbird. *Nature* **2021**, *594*, 535–540.

[2] Günther, A.; Einwich, A.; Sjulstok, E.; Feederle, R.; Bolte, P.; Koch, K.-W.; Solov'yov, I. A.; Mouritsen, H. Double-Cone Localization and Seasonal Expression Pattern Suggest a Role in Magnetoreception for European Robin Cryptochrome 4. *Curr. Biol.* **2018**, *28*, 211–223.

[3] Abramson, J.; Adler, J.; Dunger, J.; Evans, R.; Green, T.; Pritzel, A.; Ronneberger, O.; Willmore, L.; Ballard, A. J.; Bambrick, J.; Bodenstein, S. W.; Evans, D. A.; Hung, C.-C.; O'Neill, M.; Reiman, D.; Tunyasuvunakool, K.; Wu, Z.; Žemgulytė, A.; Arvaniti, E.; Beattie, C.; Bertolli, O.; Bridgland, A.; Cherepanov, A.; Congreve, M.; Cowen-Rivers, A. I.; Cowie, A.; Figurnov, M.; Fuchs, F. B.; Gladman, H.; Jain, R.; Khan, Y. A.; Low, C. M. R.; Perlin, K.; Potapenko, A.; Savy, P.; Singh, S.; Stecula, A.; Thillaisundaram, A.; Tong, C.; Yakneen, S.; Zhong, E. D.; Zielinski, M.; Žídek, A.; Bapst, V.; Kohli, P.; Jaderberg, M.; Hassabis, D.; Jumper, J. M. Accurate Structure Prediction of Biomolecular Interactions with AlphaFold 3. *Nature* **2024**, *630*, 493–500.

[4] Zoltowski, B. D.; Chelliah, Y.; Wickramaratne, A.; Jarocha, L.; Karki, N.; Xu, W.; Mouritsen, H.; Hore, P. J.; Hibbs, R. E.; Green, C. B.; Takahashi, J. S. Chemical and Structural Analysis of a Photoactive Vertebrate Cryptochrome from Pigeon. *Proc. Natl. Acad. Sci. U.S.A.* **2019**, *116*, 19449–19457.

[5] Schuhmann, F.; Ramsay, J. L.; Kattnig, D. R.; Solov'yov, I. A. Structural Rearrangements of Pigeon Cryptochrome 4 Undergoing a Complete Redox Cycle. *J. Phys. Chem. B* **2024**, *128*, 3844–3855.

[6] Olsson, M. H. M.; Søndergaard, C. R.; Rostkowski, M.; Jensen, J. H. PROPKA3: Consistent Treatment of Internal and Surface Residues in Empirical pKaPredictions. *J. Chem. Theory Comput.* **2011**, *7*, 525–537.

[7] Hopkins, C. W.; Le Grand, S.; Walker, R. C.; Roitberg, A. E. Long-Time-Step Molecular Dynamics through Hydrogen Mass Repartitioning. *J. Chem. Theory Comput.* **2015**, *11*, 1864–1874.

[8] Humphrey, W.; Dalke, A.; Schulten, K. VMD – Visual Molecular Dynamics. *J. Mol. Graph.* **1996**, *14*, 33–38.

[9] Huang, J.; Rauscher, S.; Nawrocki, G.; Ran, T.; Feig, M.; de Groot, B. L.;

Grubmüller, H.; MacKerell, A. D. CHARMM36m: An Improved Force Field for Folded and Intrinsically Disordered Proteins. *Nat. Methods* **2016**, *14*, 71–73.

[10] Best, R. B.; Zhu, X.; Shim, J.; Lopes, P. E. M.; Mittal, J.; Feig, M.; MacKerell, A. D. Optimization of the Additive CHARMM All-Atom Protein Force Field Targeting Improved Sampling of the Backbone ϕ, ψ and Side-Chain χ_1 and χ_2 Dihedral Angles. *J. Chem. Theory Comput.* **2012**, *8*, 3257–3273.

[11] MacKerell, A. D.; Bashford, D.; Bellott, M.; Dunbrack, R. L.; Evanseck, J. D.; Field, M. J.; Fischer, S.; Gao, J.; Guo, H.; Ha, S.; Joseph-McCarthy, D.; Kuchnir, L.; Kuczera, K.; Lau, F. T. K.; Mattos, C.; Michnick, S.; Ngo, T.; Nguyen, D. T.; Prodhom, B.; Reiher, W. E.; Roux, B.; Schlenkrich, M.; Smith, J. C.; Stote, R.; Straub, J.; Watanabe, M.; Wiórkiewicz-Kuczera, J.; Yin, D.; Karplus, M. All-Atom Empirical Potential for Molecular Modeling and Dynamics Studies of Proteins. *J. Phys. Chem. B* **1998**, *102*, 3586–3616.

[12] Aleksandrov, A. A Molecular Mechanics Model for Flavins. *J. Comput. Chem.* **2019**, *40*, 2834–2842.

[13] Lüdemann, G.; Solov'yov, I. A.; Kubař, T.; Elstner, M. Solvent Driving Force Ensures Fast Formation of a Persistent and Well-Separated Radical Pair in Plant Cryptochrome. *J. Am. Chem. Soc.* **2015**, *137*, 1147–1156.

[14] Solov'yov, I. A.; Domratcheva, T.; Moughal Shahi, A. R.; Schulten, K. Decrypting Cryptochrome: Revealing the Molecular Identity of the Photoactivation Reaction. *J. Am. Chem. Soc.* **2012**, *134*, 18046–18052.

[15] Phillips, J. C.; Hardy, D. J.; Maia, J. D. C.; Stone, J. E.; Ribeiro, J. V.; Bernardi, R. C.; Buch, R.; Fiorin, G.; Hénin, J.; Jiang, W.; McGreevy, R.; Melo, M. C. R.; Radak, B. K.; Skeel, R. D.; Singharoy, A.; Wang, Y.; Roux, B.; Aksimentiev, A.; Luthey-Schulten, Z.; Kalé, L. V.; Schulten, K.; Chipot, C.; Tajkhorshid, E. Scalable Molecular Dynamics on CPU and GPU Architectures with NAMD. *J. Chem. Phys.* **2020**, *153*, 044130.

[16] Phillips, J. C.; Hardy, D. J.; Maia, J. D. C.; Stone, J. E.; Ribeiro, J. V.; Bernardi, R. C.; Buch, R.; Fiorin, G.; Hénin, J.; Jiang, W.; McGreevy, R.; Melo, M. C. R.; Radak, B. K.; Skeel, R. D.; Singharoy, A.; Wang, Y.; Roux, B.; Aksimentiev, A.; Luthey-Schulten, Z.; Kalé, L. V.; Schulten, K.; Chipot, C.; Tajkhorshid, E.

Scalable Molecular Dynamics on CPU and GPU Architectures with NAMD. *Chem. Phys.* **2020**, *153*, 044130.

[17] Essmann, U.; Perera, L.; Berkowitz, M. L.; Darden, T.; Lee, H.; Pedersen, L. G. A Smooth Particle Mesh Ewald Method. *J. Chem. Phys.* **1995**, *103*, 8577–8593.

[18] Jung, J.; Kasahara, K.; Kobayashi, C.; Oshima, H.; Mori, T.; Sugita, Y. Optimized Hydrogen Mass Repartitioning Scheme Combined with Accurate Temperature/Pressure Evaluations for Thermodynamic and Kinetic Properties of Biological Systems. *J. Chem. Theory Comput.* **2021**, *17*, 5312–5321.

[19] Morrone, J. A.; Zhou, R.; Berne, B. J. Molecular Dynamics with Multiple Time Scales: How to Avoid Pitfalls. *J. Chem. Theory Comput.* **2010**, *6*, 1798–1804.

[20] Miyamoto, S.; Kollman, P. A. Settle: An Analytical Version of the SHAKE and RATTLE Algorithm for Rigid Water Models. *J. Comput. Chem.* **1992**, *13*, 952–962.

[21] Ryckaert, J.-P.; Ciccotti, G.; Berendsen, H. J. Numerical Integration of the Cartesian Equations of Motion of a System with Constraints: Molecular Dynamics of N-Alkanes. *J. Comput. Phys.* **1977**, *23*, 327–341.

[22] Martyna, G. J.; Tobias, D. J.; Klein, M. L. Constant Pressure Molecular Dynamics Algorithms. *Chem. Phys.* **1994**, *101*, 4177–4189.

[23] Feller, S. E.; Zhang, Y.; Pastor, R. W.; Brooks, B. R. Constant Pressure Molecular Dynamics Simulation: The Langevin Piston Method. *Chem. Phys.* **1995**, *103*, 4613–4621.

[24] Torrie, G. M.; Valleau, J. P. Nonphysical Sampling Distributions in Monte Carlo Free-Energy Estimation: Umbrella Sampling. *J. Comput. Phys.* **1977**, *23*, 187–199.

[25] Case, D. A.; Cerutti, D. S.; Cruzeiro, V. W. D.; Darden, T. A.; Duke, R. E.; Ghazimirsaeed, M.; Giambasu, G. M.; Giese, T. J.; Gotz, A. W.; Harris, J. A.; others Recent Developments in Amber Biomolecular Simulations. *J. Chem. Inf. Model.* **2025**, *65*, 7835–7843.

[26] Kumar, S.; Rosenberg, J. M.; Bouzida, D.; Swendsen, R. H.; Kollman, P. A. THE Weighted Histogram Analysis Method for Free-Energy Calculations on Biomolecules. I. The Method. *J. Comput. Chem.* **1992**, *13*, 1011–1021.

[27] Ganguly, A.; Thaplyal, P.; Rosta, E.; Bevilacqua, P. C.; Hammes-Schiffer, S. Quantum Mechanical/Molecular Mechanical Free Energy Simulations of the Self-Cleavage Reaction in the Hepatitis Delta Virus Ribozyme. *J. Am. Chem. Soc.* **2014**, *136*, 1483–1496.

[28] Rosta, E.; Nowotny, M.; Yang, W.; Hummer, G. Catalytic Mechanism of RNA Backbone Cleavage by Ribonuclease H from Quantum Mechanics/Molecular Mechanics Simulations. *J. Am. Chem. Soc.* **2011**, *133*, 8934–8941.

[29] Reinhardt, C. R.; Sayfutyarova, E. R.; Zhong, J.; Hammes-Schiffer, S. Glutamate mediates proton-coupled electron transfer between tyrosines 730 and 731 in Escherichia coli ribonucleotide reductase. *J. Am. Chem. Soc.* **2021**, *143*, 6054–6059.

[30] Götz, A. W.; Clark, M. A.; Walker, R. C. An Extensible Interface for QM/MM Molecular Dynamics Simulations with AMBER. *J. Comput. Chem.* **2014**, *35*, 95–108.

[31] Wong, S. Y.; Wei, Y.; Mouritsen, H.; Solov'yov, I. A.; Hore, P. J. Cryptochrome Magnetoreception: Four Tryptophans Could be Better Than Three. *J. R. Soc. Interface* **2021**, *18*, 20210601.

[32] Timmer, D.; Frederiksen, A.; Lünemann, D. C.; Thomas, A. R.; Xu, J.; Bartölke, R.; Schmidt, J.; Kubar, T.; De Sio, A.; Solov'yov, I. A.; Mouritsen, H.; Lienau, C. Tracking the Electron Transfer Cascade in European Robin Cryptochrome 4 Mutants. *J. Am. Chem. Soc.* **2023**, *145*, 11566–11578.

[33] Luo, J.; Hungerland, J.; Solov'yov, I. A.; Subotnik, J. E.; Hammes-Schiffer, S. Protein and Solvent Reorganization Drives Radical Pair Stability in Avian Cryptochrome 4a. *J. Am. Chem. Soc.* **2025**, *147*, 43934–43945.

[34] Soudackov, A.; Hammes-Schiffer, S. Derivation of Rate Expressions for Nonadiabatic Proton-Coupled Electron Transfer Reactions in Solution. *J. Chem. Phys.* **2000**, *113*, 2385–2396.

[35] Hammes-Schiffer, S. Proton-Coupled Electron Transfer: Moving Together and Charging Forward. *J. Am. Chem. Soc.* **2015**, *137*, 8860–8871.
